# A food bank program to help food pantries improve healthy food choices: mixed methods evaluation of The Greater Boston Food Bank’s Healthy Pantry Program

**DOI:** 10.1186/s12889-023-15243-4

**Published:** 2023-02-17

**Authors:** Jenny Jia, Rachel Burgun, Alexa Reilly, Ross Sonnenblick, Lauren Fiechtner, Rachel M. Zack, Bianca Porneala, Anne N. Thorndike

**Affiliations:** 1 Division of General Internal Medicine, Massachusetts General Hospital, Boston, MA USA; 2 Division of General Internal Medicine, Department of Medicine, Northwestern University, 750 Lakeshore Drive, 10th floor, Chicago, IL 60611 USA; 3 The Greater Boston Food Bank, Boston, MA USA; 4 Division of General Academic Pediatrics and Division of Gastroenterology and Nutrition, Mass General Hospital for Children, Boston, MA USA; 5 Harvard Medical School, Boston, MA USA

**Keywords:** Food pantries, Charitable food system, Nudge interventions, Healthy food choices

## Abstract

**Background:**

The Greater Boston Food Bank’s (GBFB) Healthy Pantry Program (HPP) is an online training that teaches food pantry staff to implement behavioral nudges (e.g., traffic-light nutrition labels, choice architecture) to promote healthier client choices. This study assessed if HPP was associated with healthier food bank orders by food pantries and identified implementation facilitators and barriers.

**Methods:**

This mixed methods study collected quantitative data from a matched cohort of 10 HPP food pantries and 99 matched control food pantries in eastern Massachusetts that allow clients to choose their own food, and qualitative data from structured individual interviews with 8 HPP pantry staff. A difference-in-differences analysis compared changes in percentage of pantries’ food bank orders (by weight) of foods labeled green/yellow (healthier choices) and fresh produce from baseline to 6 and 10 months between HPP and control pantries. Interviews were coded for implementation facilitators and barriers.

**Results:**

Before starting HPP, green-yellow ordering was 92.0% (SD 4.9) in control and 87.4% (SD 5.4) in HPP pantries. Participation in HPP was not associated with changes in green-yellow or fresh produce ordering at 6 or 10 months. HPP implementation facilitators included HPP training being accessible (sub-themes: customizable, motivating) and compatible with client-choice values. Barriers included resource limitations (sub-themes: staff shortage, limited space) and concerns about stigmatizing client food choices with use of labels for unhealthy foods.

**Conclusions:**

An online program to help pantries promote healthier client choices was not associated with changes in how much healthy food pantries ordered from the food bank, suggesting it did not substantially change client choices. Implementation challenges and high baseline healthy ordering may have influenced HPP’s effectiveness.

**Supplementary Information:**

The online version contains supplementary material available at 10.1186/s12889-023-15243-4.

## Introduction

Food insecurity affects approximately one in ten Americans and is associated with poor nutrition, diabetes, hypertension, and cardiovascular diseases [1, 2]. Approximately a quarter of food-insecure households in the U.S. report using food pantries [3]. Although some pantries are making efforts to stock healthier foods, groceries offered at food pantries often lack the nutritional value necessary to support healthy diets [4]. Prior surveys have demonstrated that clients consider healthy foods as one of the most important pantry priorities [5, 6]. Evidence-based strategies to change the nutritional quality of available foods and promote selection of healthier foods by food pantry clients are important tools to mitigate adverse health outcomes in food-insecure households.

Behavioral nudges are alterations in the context in which people make decisions (e.g., grocery stores, cafeterias) to create a predictable change in human behavior without affecting available options and economic incentives [7]. Nudging strategies have been effective in promoting healthy food choices in workplace cafeterias and retail settings [8–12]. Food pantries are increasingly interested in incorporating behavioral nudge interventions to encourage healthier food selection by pantry clients [13], but interventions generally have limited scope, and few programs undergo rigorous evaluation. Two observational studies using nudges in food pantries saw improvements in client selection of healthier foods [14, 15], though one was limited to a single healthy shelf within a pantry. Prominent placement of specific foods and bundle interventions, where ingredients used to cook a healthy dish are grouped together, increased healthy food selection in food pantries [16–18]. Pilot pre-post studies on traffic-light labeling, where foods are labeled green, yellow, or red based on their nutrition value with green indicating the healthiest foods, have shown increases in healthy food choices by clients in food pantries [19, 20]. In addition, traffic-light interventions in food bank inventory platforms through which food pantries order groceries are associated with increases in procurement of green-labeled foods by food pantries [21, 22].

This study evaluated the implementation and effectiveness of a food bank-led program targeting food pantry staff that teaches behavioral economic strategies to pantry staff to improve client selection of healthy foods. Healthy Pantry Program (HPP) is an online training program developed and implemented by The Greater Boston Food Bank (GBFB) for pantry staff to learn how to use a traffic-light nutrition ranking system and a multilingual healthy recipe website and how to implement behavioral nudges. This study evaluated whether food pantries participating in HPP increased the proportion of healthy foods ordered from GBFB more than food pantries that had not participated in HPP, as a proxy for measuring changes in pantry client healthy food selection. Qualitative interviews with HPP pantry staff were conducted to identify facilitators and barriers to program implementation that could inform quantitative results. The hypothesis was that HPP pantries would increase their orders of healthier foods from GBFB compared to matched control pantries.

## Methods

This mixed-methods study to evaluate HPP included a secondary analysis of previously collected quantitative data augmented by qualitative interviews of pantry staff who participated in HPP. Quantitative data was analyzed prior to qualitative data analysis, and results of both study components were integrated following the qualitative analysis. This study was approved by the Mass General Brigham (formerly “Partners”) Institutional Review Board. It was funded by a pilot grant from GBFB, and employees of GBFB participated as co-authors.

### Study setting and target population

GBFB is a regional food bank located in eastern Massachusetts and is the largest hunger-relief organization in New England. It supplies grocery staples to 600 food distribution partners including 365 food pantries, of which 311 have client-choice operating models (i.e., clients have some level of choice over the groceries they take from pantry shelves or a menu) [23]. GBFB-partnered food pantries in eastern Massachusetts order groceries, including foods, beverages, toiletries, and other household essentials, from GBFB. The majority of products, including all fresh produce, are free of cost to partner agencies. Additionally, GBFB provides a co-op, pass-through program where food pantries can purchase some items at lower than market cost. GBFB has developed policies and implemented systems that prioritize the acquisition of nutrient dense foods. Since January 2018, GBFB has included traffic-light labels using the Supporting Wellness at Pantries (SWAP) nutrition ranking system on the pantry ordering platform to help pantry staff and volunteers identify healthier options [24]. SWAP ranks food as green (“Choose often”), yellow (“Choose sometimes”), or red (“Choose rarely”) according to sugar, saturated fat, and sodium content (Additional file 1) [24]. It was designed to be user-friendly for pantry staff and volunteers who may not have training in nutrition. GBFB also facilitates partnerships between food pantries and local grocery stores for direct store-to-pantry donations. Food pantries may also obtain groceries through donations from local farms and community food drives and purchases from grocery stores or wholesalers.

Pantries eligible for this study were client-choice food pantries partnered with GBFB. Client-choice status was self-reported and varied in degree from a basic level, where clients receive pre-packaged bags but may choose a select number of items, to a maximum level, where clients shop from a pantry’s entire inventory without any restrictions on choice or quantity, akin to a grocery store. A summary score was developed by GBFB to categorize affiliated food pantries into tiers using a weighted score that incorporates pantry characteristics, such as annual food distribution by weight, average clients per month, storage capacity, pantry hours, and frequency of operations. From 2018 to 2019, ten client-choice food pantries participated in HPP. GBFB initially recruited 5 food pantries to pilot the first iteration of HPP in October 2018, which balanced an adequate number of pilot pantries with the GBFB team’s implementation capacity. These 5 sites were recommended by community-engaged GBFB staff, and GBFB used feedback from wave 1 pantry staff after completing HPP to make improvements to the program. The second wave of 5 pantries piloted the next iteration of HPP. This wave included pantries that reached out to the registered dietician nutritionist (RDN) after seeing or hearing information about HPP and pantries that had indicated interest in nutrition training via a GBFB annual survey. In May 2019, wave 2 pantries began the second iteration of HPP, which had minor changes, such as eliminating site visits and adding a learning management system, to decrease the RDN’s involvement and promote scalability. Control pantries were eligible pantries that had never participated in HPP and were matched to 1 or more HPP pantries by summary score deciles and baseline green-yellow food ordering. Monthly green-yellow food ordering was matched within 10 percentage points for 12-month baseline orders and 5 percentage points for 3-month baseline orders.

Eligible pantry staff for the qualitative study were staff of the 10 HPP pantries who had participated in HPP training in 2018 or 2019. Since the pool of eligible participants was small (*n* = 15), reaching thematic saturation was not assumed. Each eligible participant received up to 3 emails from the RDN at GBFB, followed by a phone call if there was no response. The RDN had a prior working relationship with eligible pantry staff through HPP and other GBFB programming initiatives. The RDN’s recruitment emails included research staff that coordinated interviews with eligible individuals who responded. The research staff had no prior relationship with participants. Interviewees received a $50 gift card for their time.

### Healthy Pantry Program

HPP was created by a RDN (RB) at GBFB to meet the demand for nutrition training from pantry staff. The program training consisted of multimedia educational lessons organized into three 1-hour modules: the SWAP traffic-light nutrition labeling [24]; a multilingual healthy recipe website called Click ‘N Cook; and education on 7 different types of client-facing nudges that could be implemented in the physical pantry space. The version of SWAP that was used in this training used the initial saturated fat, sodium, and total sugars thresholds; SWAP was revised in 2020 with updated nutrition thresholds [25]. HPP teaches pantry staff that both green- and yellow-labeled foods are preferred to red-labeled foods. Click ‘N Cook was developed internally by GBFB and contains only green- and yellow-labeled recipes with traffic-light labels for each recipe visible to website visitors. It is available in Arabic, Chinese (simplified), English, Haitian Creole, Portuguese, Russian, and Spanish. The 7 HPP nudge interventions were based on previous successful studies on nudges in the retail setting: [12] “front and center placement” (e.g., items at eye level), “multiple exposure” (e.g., items displayed in multiple places), “abundance” (e.g., displays suggesting there are many of the item available), “display change” (e.g., items placed in attractive bins), “bundling and recipes” (e.g., recipe card next to item), “priming” (e.g., posters at entrance to get clients to think about the target item before shopping), and “shelf tags” (e.g., SWAP traffic-light ranking). All HPP pantries also received materials to improve the client experience: traffic-light labels (green, yellow, and red in wave 1; green and yellow only in wave 2), signage, posters, shelving, food baskets, and lighting. GBFB decided to stop providing red labels to wave 2 pantries after informal conversations with wave 1 pantry staff and meetings with other food banks regarding concern over stigma associated with labeling foods red. In wave 1, the GBFB RDN emailed each online module to staff members upon completion of the prior module, and in wave 2, staff accessed the modules via a learning management system. Pantries were given 3 months to complete HPP training. Finally, in wave 1, HPP included a site visit with the GBFB RDN (RB) after completion of the online training to provide pantry-specific recommendations on what components to implement. Four of 5 HPP pantries in wave 1 completed the site visit. In wave 2, site visits were not provided, though 1 HPP pantry requested and was granted a RDN site visit, which included the same content as wave 1 site visits.

### Quantitative outcome measures

Pantry ordering data came from GBFB’s NetSuite database, which included the weight in pounds of monthly total orders, SWAP-ranked foods, and SWAP green/yellow/red-labeled foods, as well as weights, food category (e.g., fresh produce, salvage), and SWAP ranking for specific products ordered. Each study outcome was a percentage that was calculated from the weight ordered of the food of interest divided by the total weight of SWAP-ranked foods ordered per pantry per month. For example, a food pantry in October 2017 that ordered 915 lbs. of green foods, 770 lbs. of yellow foods, and 2125 lbs. of ranked foods had a green-yellow ordering of (915 + 770)/2125 = 87.9% for that month. Products that were not assigned a SWAP ranking by GBFB were excluded, such as a variety of different cereals pooled from donations that would not all be the same SWAP color, which was approximately 5% by weight of total products distributed by GBFB.

The 12-month baseline period was from October 2017 to September 2018 for wave 1 HPP and control pantries and May 2018 to April 2019 for wave 2 HPP and control pantries. The primary outcome in this study was change in pantry green-yellow GBFB ordering from 12-month baseline to 6 months (0 to 6 months) and 10 months (7 to 10 months) after the start of the program. This composite outcome was chosen because the HPP training taught pantry staff that both green and yellow foods were healthy compared to red foods. Secondary outcomes included change in green ordering, yellow ordering, and fresh produce ordering.

### Statistical analysis

Descriptive statistics were generated for pantry characteristics with continuous variables reported as means with standard deviations and categorial variables as frequency counts with percentages. Longitudinal mixed-effects models were used to calculate difference-in-difference estimates in study outcomes in HPP pantries compared to control pantries from baseline to follow-up. As seasonality often affects food supply in the charitable food system, models were adjusted for seasonality using dummy variables for each season: spring (March–May), summer (June–August), fall (September–November), and winter (December–February).

#### Qualitative methods

### Data collection

Structured interviews were conducted over a web-based video conferencing platform. Participants were interviewed individually and once, except for 2 participants from the same site who requested and were granted an interview together. To ensure confidentiality, all clinical research staff and interviewees attended the video calls from private settings. Consent forms were emailed to interviewees by research staff prior to their interviews, and verbal consent was obtained at the beginning of each interview. Interviewees were asked a series of free-response questions, with additional probing questions as necessary. Interviews lasted approximately 45 minutes and were conducted by clinical research staff (AR, clinical research coordinator, female, prior experience interviewing; RS, clinical research coordinator, male, no prior experience; or JJ, principal investigator, female, prior experience interviewing and analyzing qualitative data) with the GBFB RDN (RB) present and available as a resource for the interviewer and participant; no fieldnotes were taken. Participants did not see transcripts or provide further feedback after interviews.

Interview questions were adapted from the Consolidated Framework for Implementation Research (CFIR) questionnaire [26] and were not adjusted or changed throughout the qualitative data collection process. CFIR highlights five categories of constructs that are associated with effective implementation of interventions: intervention characteristics (e.g., cost, adaptability), outer setting (e.g., needs of those served by the organization, networking with other organizations), inner setting (e.g., organizational culture, communication), individual characteristics (e.g., thoughts and beliefs of stakeholders), and process (e.g., stakeholder engagement). The interview questionnaire included demographic information and questions covering CFIR domains, HPP components, and pantry operations (Additional file 2). It was piloted with 2 separate GBFB staff.

### Data analysis

After conducting all study interviews, audio recordings of interviews were transcribed by a professional transcription service, and transcripts were analyzed using Dedoose (v 9.0.17, Los Angeles, CA) [27]. The coding scheme was generated a priori and consisted of CFIR constructs and HPP implementation codes (e.g., traffic light labeling implementation). Many codes contained subcodes, and the most specific code was selected in each case. Excerpts were assigned multiple codes when applicable. Each interview was independently coded by 2 research staff (AR, RS). Both versions of each coded transcript were reviewed, and discrepancies were resolved through discussion between both coders and the principal investigator (JJ). Coded excerpts were organized by code under the CFIR major constructs (Intervention Characteristics, Outer Setting, Inner Setting, Characteristics of Individuals, and Process). The research coding team (AR, RS, JJ) held weekly meetings to review excerpts under each major construct en bloc to identify facilitator and barrier themes within each construct. Relationships between themes were considered to reorganize certain themes into subthemes within a larger, common theme (e.g., the themes lack of physical space and lack of staff time could become subthemes under a new, broader theme “lack of resources”).

## Results

### Quantitative

The pantry sample included all 10 client-choice pantries that participated in HPP and all controls (*n* = 99) matched using the pantry summary score and baseline pantry orders from a pool of 298 client-choice pantries in the GBFB network that did not participate in HPP during the study period. HPP pantries generally served fewer clients per month (HPP mean = 657 clients, SD 798; control mean = 1439 clients, SD 2173) and distributed less food by weight each year (HPP mean = 130,000 lbs., SD 195,000; control mean = 273,000 lbs., SD 393,000) compared to control pantries (Table 1). Having a maximum client-choice operation model was more highly represented in HPP pantries (70.0%) than in control pantries (43.3%). Nine of 10 HPP pantries had at least 1 staff member complete all 3 training modules; 1 HPP pantry finished 2 of 3 modules. Seven HPP pantries had 1 staff member participate in HPP training, 2 HPP pantries had 2 participating staff members, and 1 HPP pantry had 3 participating staff members.

**Table 1:** Characteristics of Healthy Pantry Program (HPP) and control pantries food pantries from eastern Massachusetts

	All ***n*** = 109	HPP ***n*** = 10	Control ***n*** = 99
Clients served per month, mean (SD)	1367 (2096)	657 (798)	1439 (2173)
Distributed food, 1000 lb./yr, mean (SD)	288 (405)	130 (195)	273 (393)
Client choice, n (%)			
Maximum	50 (45.9)	7 (70.0)	43 (43.3)
Moderate	24 (22.0)	1 (10.0)	23 (23.2)
Basic	22 (20.2)	1 (10.0)	21 (21.2)
Menu	13 (11.9)	1 (10.0)	12 (12.1)
Pantry catchment area, n (%)			
Restricted to town/city of pantry	79 (72.5)	6 (60.0)	73 (73.7)
No geographic restrictions	30 (27.5)	4 (40.0)	26 (26.3)
Permitted client visits per month, n (%)			
> 2 times	23 (21.1)	2 (20.0)	21 (21.2)
2 times	24 (22.0)	3 (30.0)	21 (21.2)
1 time	62 (56.9)	5 (50.0)	57 (57.6)
Cooler capacity, n (%)			
Very small/small	40 (36.7)	4 (40.0)	36 (36.3)
Medium	20 (18.3)	3 (30.0)	17 (17.2)
Large	19 (17.4)	2 (20.0)	17 (17.2)
Very large	24 (22.0)	1 (10.0)	23 (23.2)
None	6 (5.5)	–	6 (6.1)
Freezer capacity, n (%)			
Very small/small	20 (18.4)	3 (30.0)	17 (17.2)
Medium	28 (25.7)	1 (10.0)	27 (27.3)
Large	20 (18.3)	3 (30.0)	17 (17.2)
Very large/extra large	40 (36.7)	3 (30.0)	17 (17.2)
Extra large			20 (20.2)
None	1 (0.9)	–	1 (1.0)

**Fig. 1:**
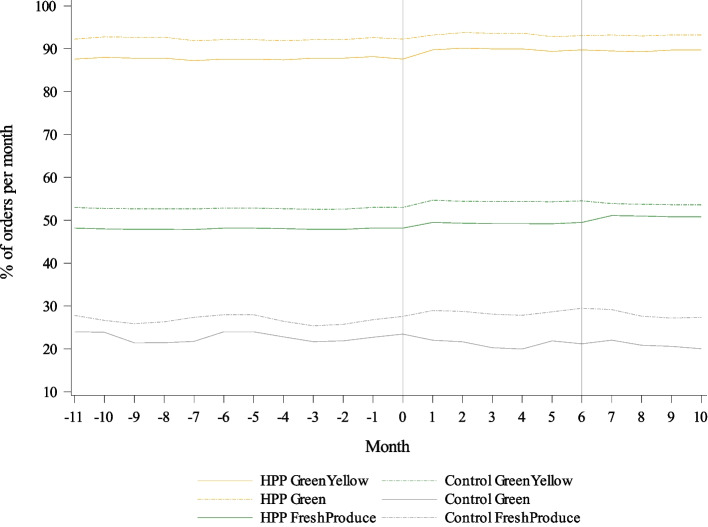
Trends in green-yellow, green only, and fresh produce ordering as percentages of total pounds of monthly pantry orders by Healthy Pantry Program (HPP) and control groups. The x-axis shows time in months with month 0 indicating the start of the program. The y-axis shows food orders by pantries from the food bank as a percentage of total pounds of food ordered per month (range 10 to 100%). Analyses were adjusted for seasonality

Over the study period, green-yellow and green only pantry ordering were stable in both the HPP and control groups, while fresh produce ordering diverged slightly, with slight decreases in fresh produce ordering as a percentage of total pounds of food ordered in the HPP group (Fig. 1). In this sample, 12-month baseline mean green-yellow ordering was 92.0% (SD 4.9) in control pantries and 87.4% (SD 5.4) in HPP pantries. The change in green-yellow ordering for HPP pantries was 0.9 percentage points (95% CI -2.5, 4.2) higher at 6 months and 0.7 percentage points (95% CI -3.4, 4.8) higher at 10 months than for control pantries, adjusted for seasonality (Table 2). When separated into individual traffic-light colors, changes in yellow ordering associated with HPP were 2.7 percentage points (− 4.2, 9.6) greater at 6 months and 0.8 percentage points (− 8.5, 6.9) less at 10 months, and changes in green ordering were associated with HPP, which were 1.9 percentage points (− 8.9, 5.0) less at 6 months and 1.8 percentage points (− 6.1, 9.6) greater at 10 months. Twelve-month mean fresh produce ordering at baseline was 28.4% (SD 14.1) of all GBFB orders in control pantries and 22.3% (SD 11.9) in HPP pantries. HPP was associated with a decrease of 4.0 percentage points (95% CI -11.1, 3.1) in fresh produce ordering at 6 months and a decrease of 4.6 percentage points (95% CI -12.4, 3.3) at 10 months.

**Table 2:** Unadjusted baseline means and seasonality-adjusted difference-in-difference estimates in green-yellow, green only, yellow only, and fresh produce ordering as percentages of total weight in pounds of monthly pantry orders between Healthy Pantry Program (HPP) and control pantries

	Baseline Mean (SD)	6-month difference Mean (SD)	6-month difference-in-difference (95% CI)	10-month difference Mean (SD)	10-month difference-in-difference (95% CI)
% Green-yellow food orders					
HPP	87.5 (5.5)	1.7 (3.2)	0.9 (−2.5, 4.2)	1.3 (3.9)	0.7 (−3.4, 4.8)
Control	92.1 (4.8)	0.8 (1.0)		0.6 (1.2)	
% Yellow food orders					
HPP	39.2 (9.0)	1.9 (6.5)	2.7 (−4.2, 9.6)	1.0 (7.3)	−0.8 (−8.5, 6.9)
Control	38.7 (10.0)	−0.8 (2.0)		1.8 (2.3)	
% Green only food orders					
HPP	48.3 (8.1)	0.2 (6.6)	−1.9 (−8.9, 5.0)	1.0 (7.4)	1.8 (−6.1, 9.6)
Control	53.5 (11.4)	2.2 (2.1)		−0.8 (2.3)	
% Fresh produce					
HPP	22.4 (12.1)	−1.7 (6.7)	−4.0 (− 11.1, 3.1)	−2.5 (7.3)	−4.6 (− 12.4, 3.3)
Control	28.1 (13.9)	2.3 (2.2)		2.1 (2.6)	

### Qualitative

All 15 pantry staff who participated in HPP training from 2018 to 2019 were contacted by email; 1 email address resulted in an automatic reply that the staff member had left the organization, 1 individual replied that she no longer worked there, and 5 did not respond. Eight pantry staff representing 6 HPP pantries (4 who started HPP in October 2018 and 2 who started in May 2019) were enrolled. Of the 8 pantry staff members, 3 interviewees (37.5%) were paid staff and worked at 2 (33.3%) of the 6 HPP pantries involved in the qualitative study (Additional file 3). Of the 6 HPP pantries involved in the qualitative study, 5 (83.3%) served fewer than 500 clients per month on average, while 1(16.7%) served more than 1000 clients per month on average.

Interviewees reported that they implemented some of the HPP components in their pantries. Staff from 2 pantries (33.3%) reported using client-facing traffic-light labels, and staff from 5 pantries (83.3%) reported using 1 or more of the 7 nudges. Staff from all 6 pantries (100.0%) reported using the Click ‘N Cook healthy recipe website. In addition, staff from 5 pantries (83.3%) said that they took into consideration the traffic-light colors of foods when ordering from GBFB’s online platform.

### Implementation facilitators

Pantry staff described multiple facilitators to implementation of HPP in their pantries. Major themes in facilitators were 1.) HPP was aligned with organizational values and 2.) HPP training was accessible, with subthemes of the training being customizable and motivating. The majority of staff interviewees thought the choice architecture premise of HPP was highly compatible with the values of client-choice pantries, namely maintaining choice while promoting healthy lifestyles (Table 3, Quote 1). Interviewees generally agreed that the HPP training delivered content in a manner that was useful, sufficient, and customizable; moreover, they agreed that the training empowered staff to feel able to implement HPP components (Table 3, Quotes 2 & 3). Finally, though staff did not initially identify any external incentives for implementing HPP, a minor theme identified was that the program was an engaging discussion point with potential donors for 1 pantry (Table 3, Quote 4).

**Table 3:** Themes and representative quotes from structured interviews with 8 food pantry staff from 6 pantries on the implementation of Healthy Pantry Program (HPP)

Themes	Sub-themes and Quotes
**Facilitators**	
HPP aligned well with the values and culture of client-choice food pantries	1. *“We …have a model where our clients shop and have 100% choice…So when we were presented with the opportunity for a Healthy Pantry, for us, that really aligned with our mission of being a good resource that would improve the quality of life for our clients.”*
HPP training was accessible to pantry staff	2.Customizable: *“So having it broken up the way it was with the SWAP scores, with nudges, with Click and Cook really allowed that progression…It has tremendous depth with regard to the levels that people can go to with it. You can make very minor choices. You can make moderate choices. You can make completely revolutionary choices all within the program.”* 3.Motivating: *“I could watch a module and say, “I could probably do something like this this week.”*
HPP can engage potential donors	4. *“[Healthy Pantry Program] was a story line that we were able to talk about and a new aspect of our existing program; I think it gave us some great talking points to new potential funders, to our community members.”*
**Barriers**	
HPP implementation requires substantial resources	5. *Staff time: “Funding is a limitation on how much extra time employees can be kept working on other projects at the pantry…The Healthy Pantry Program definitely went by the wayside at some point in time.”* 6. *Staff time: “We would sometimes get an ingredient right before we’re about to open for the day, which doesn’t allow us a whole lot of time to print something up to get ready to present a recipe for that ingredient or to figure out what the nutrition values are.”* 7. *Pantry space: “I would have liked to have been able to move my signs around more easily, and have more space, in terms of having the individual items better labeled. But my pantry didn’t help. The space is limiting.”* 8. *Pantry space: “Well, it’s a very tight fit in our pantry…One of the pluses was this little shelf thing [we had] was actually somewhat in people’s way. So when they came in with their cart, they sort of had to stand right next to it…the shopper helper would be right there next to it saying like, ‘Oh, you’re waiting here. Oh, look what’s here this weekend.’”*
Pantry staff are concerned about increasing stigma towards clients	9. *“We had started to talk about what to do about red dots… we’re hoping to serve them things that they love and want to eat and have and not tell them, ‘No, no, no, you can’t have a bag of [that].’”*
**Staff Suggestions**	
Increase follow-up technical assistance after HPP training	10. *“I do then remember meeting with [the GBFB dietitian] a couple of different times at our pantry where [she] provided feedback on different opportunities…really specific recommendations around physically moving around certain food items in order to create healthier options at eye level.”*
	11. *“The only module I might suggest would be a follow-up module from Healthy Pantries who have gone through some of the training and implementation so that they can share best practices that they discovered with implementation.”*
Involve staff members who control pantry food supply (e.g., GBFB orders, donations)	12. *“The person that should be involved is the purchaser or the person who’s obtaining the food because you’re only as good as what comes in…People donate a vast quantity of all sorts of stuff.”*
Designate multiple lead implementers per site	13. *“It was kind of motivating to have a buddy because we’d be like, ‘Are you done with the course? What’s your project?’ …So it’s nice to have another person that you’re thinking about this with.”*

### Implementation barriers

Staff also identified major themes in barriers to implementing HPP, including 1.) resource limitations (sub-themes of limited staff time and space shortage) and 2.) concern about increasing client stigma in the pantry setting. Pantry staff judged the material costs of implementing HPP components to be low (e.g., cost of display baskets, printing recipes), but they frequently cited lack of staff time for implementation and sustainability of HPP components (Table 3, Quotes 5 & 6). In addition, staff mentioned lack of pantry space as a hindrance to implementing HPP components as designed. Space limitations could include a small pantry footprint, lack of shelf space to place traffic-light labels, or mixed-use space that required setting up and dismantling the pantry regularly (Table 3, Quote 7). However, in 1 HPP pantry, limited space led to a positive opportunity for pantry staff to engage with clients on healthy options at the pantry using a cart that partially obstructed the flow of clients through the physical space (Table 3, Quote 8). Lastly, some interviewees expressed concern about judgment or stigma inadvertently created by certain HPP components, especially how clients would feel seeing red labels discouraging them from choosing certain options (Table 3, Quote 9).

Suggestions to improve HPP included minor themes of 1.) follow-up training after HPP core training was complete and 2.) additional pantry personnel who should be involved. Some wave 1 HPP pantries that received a GBFB dietitian visit after completing the online course endorsed the usefulness of this program component (Table 3, Quote 10), and others suggested learning groups for all pantries participating in HPP to share best practices while implementing HPP (Table 3, Quote 11). In addition, interviewees recommended that each pantry have more than one key HPP implementer and that key implementers include personnel responsible for pantry supply (Table 3, Quotes 12 & 13). Food supply staff were favored because they could order healthier foods from GBFB or could encourage community donations to become healthier.

## Discussion

In this mixed methods evaluation, there was no evidence that HPP was associated with significant changes in pantry ordering of healthy food from the food bank. Qualitative interviews with pantry staff revealed several barriers to successful implementation of the program. Though pantry staff viewed HPP as closely aligned with the values of client-choice pantries and viewed its associated training as empowering and accessible, limited resources (e.g., staff time and pantry space) and concerns of increasing client stigma hindered the implementation of HPP.

The qualitative findings of difficulty with implementation may help explain why HPP pantries did not increase healthy food orders compared to the control pantries. Prior research has demonstrated that traffic-light labeling and choice architecture are promising strategies to promote healthier food choices in food pantries; however, research has also shown that the degree of implementation affects outcomes and that shifts in client choices may not be reflected in pantry-level inventory. In a pilot study, SWAP traffic-light labeling incorporated into one pantry led to decreases in red food selection by clients [19]. Another pilot study also found increased healthy food selection with client-facing SWAP labels despite no changes detected in the pantry inventory [20]. An evaluation of SuperShelf, a multicomponent pantry intervention that incorporated food supply change and choice architecture, included 2 intervention pantries and found improvements in the dietary quality of pantry baskets in the intervention pantry with high fidelity to the intervention but no changes in the intervention pantry with low fidelity. In this study, the lack of changes seen in pantry orders may have been due to variation in the implementation of the behavioral nudges and variable uptake of client-facing traffic-light labeling.

The relatively high baseline percentages of green and yellow food orders for all GBFB pantries may have had ceiling effects and contributed to the lack of improvement in HPP study outcomes. Coexisting programs from the GBFB also incentivized nutritious food in pantries during the study period. The HPP training taught food pantries that green and yellow foods were healthier than red foods but did not explicitly distinguish between green and yellow. Future interventions incorporating traffic-light nutrition labeling in charitable food settings with high baseline healthy food distribution could emphasize green foods over yellow. In addition, food banks and pantries may consider setting organizational goals to limit red foods.

HPP pantries generally viewed HPP as aligned with common organizational goals of supporting the health of clients without compromising their ability to choose. The ability to make choices is one of the most important pantry factors identified by clients [5]. The behavioral strategies used in HPP likely promoted buy-in among client-choice pantries since maintaining choice was one of highest priorities of these pantries. In particular, red traffic-light labels elicited concerns from pantry staff about increased perceptions of judgment or stigma among pantry clients about choosing red-labeled foods, which was also captured in a prior study of food pantry staff [28]. These concerns were significant enough that one pantry in wave 1 elected to use only green and yellow labels when red were also provided, and GBFB did not distribute red labels to wave 2 HPP pantries due to the concern of stigma. Traffic-light labeling has shown success in various for-profit settings, [9, 10, 29, 30] with some research suggesting that red labels are more effective than green labels in changing behavior [31]. However, in certain settings or special populations, such as food pantries where clients experience more stigma, a program’s effectiveness must be balanced with its appropriateness for the implementation context. Pantry client feedback on traffic-light labeling is limited to survey data that does not clearly indicate whether pantry clients perceive it as appropriate for the food pantry setting. One study found overwhelming support for SWAP labels (95%) among surveyed clients in a rural food pantry, [32] but another found that clients were neutral on traffic-light labeling and were more supportive of other nutrition interventions in four pantries in Connecticut [6]. As client-facing SWAP labels are increasingly being incorporated into the food pantry space, research is needed on how pantry clients perceive this type of intervention, both its positive and negative aspects, and whether it needs augmentation to be equitable and useful to clients.

Staff interviewees thought that the information provided in the HPP training modules was accessible and presented in a way that motivated the staff member to action. The organization of components into modules provided a sense of customizability that pantry staff appreciated, especially due to the heterogeneity of food pantries, including variations in staff capacity, space considerations, and clientele. Though the training offered sufficient and useful information to implement HPP components, the intervention may have benefitted from augmentation with follow-up technical assistance, especially after completion of all HPP modules, such as learning groups, virtual or in-person site visits by GBFB, or best-practices dissemination from previous participating pantries. The non-uniformity in implementation of RDN site visits within and between the two waves of HPP limited the ability to assess the influence of site visits on study outcomes. Factors that improve intervention sustainment include maintaining workforce skills using continued or booster trainings. This suggests that more follow-up engagement could improve program implementation and sustainability [33].

This study has multiple limitations. HPP was not designed or implemented as a research study; there were important differences in the implementation of HPP between wave 1 and 2 (e.g., RDN emailed each module to staff vs. staff accessed module through a learning management system) and between pantries within wave 2 (e.g., one pantry requested and received a RDN visit). Therefore, results should be interpreted with caution. It also used previously collected pantry-level ordering data to create a matched cohort study. Despite matching pantries, there is still likely residual confounding through the use of a pantry summary score instead of direct matching on pantry characteristics and characteristics for which no data existed. However, this study demonstrates one way in which regional charitable food systems can leverage existing data for pragmatic evaluations. A limitation of the qualitative data collection was that not all interviews were conducted with individual participants; two staff members from one pantry were interviewed together. Furthermore, the quantitative and qualitative study did not capture the behaviors of individual clients at the pantry or their perceptions before and after HPP was implemented. Due to the small target population and sample size of interviewees, this study may not have reached thematic saturation in the qualitative data. In addition, transcripts were not fully checked against recordings or reviewed by interviewees, which could affect the data quality and interpretation. Finally, the food pantries from this study setting may not reflect food pantries across other regions of the US, which limits the generalizability of these findings. It is possible that in regions where food pantry orders are less healthy, such interventions could be more effective.

## Conclusion

A food bank online program to teach food pantry staff how to implement SWAP traffic-light labels, behavioral nudges, and healthy recipes in the food pantry space to promote healthy food selection by clients was not associated with changes in the healthfulness of food pantry orders from the food bank. Food pantry staff who participated in the program thought it was highly compatible with client-choice values, presented information that was accessible and motivating, and created programming that was attractive to food pantry donors. However, they cited resource limitations with staff time and space as well as concerns over increasing stigma towards clients as barriers to implementation. The lack of changes in pantry food ordering associated with the program may be partially explained by suboptimal implementation among HPP pantries, high baseline healthy food orders, and the possibility that client-level changes were not reflected in pantry-level changes. As the charitable food system shifts towards supporting nutrition and long-term health of food-insecure Americans, further research to improve dissemination, implementation, and effectiveness of nutrition programs in food pantries is needed.

## Supplementary Information

**Additional file 1.** Nutrition thresholds of green, yellow, red categories for various food groups in the 2019 Supporting Wellness at Pantries (SWAP) traffic-light nutrition ranking system. A graphic containing thresholds of saturated fat, sodium, and sugar content by food group to categorize foods into green (choose often), yellow (choose sometimes), or red (choose rarely) in the Supporting Wellness at Pantries system.

**Additional file 2.** Interview questionnaire administered to food pantry staff who participated in Healthy Pantry Program training. Document of interview questionnaire used for pantry staff interviews with CFIR domains.

**Additional file 3.** Demographic and work characteristics of pantry staff interviewees who had participated in Healthy Pantry Program training. A table of descriptive data on the pantry staff who were interviewed for this study.

## Data Availability

The datasets during and/or analyzed during the current study are available from the corresponding author on reasonable request.

## References

[1] Household food security in the United States in 2020 [https://www.ers.usda.gov/webdocs/publications/102076/err-298.pdf?v=6888.9].

[2] Gundersen C, Ziliak JP (2015). Food insecurity and health outcomes. Health Aff.

[3] Coleman-Jensen A, Rabbitt MP, Gregory CA, Singh A (2018). Statistical supplement to household food security in the United States in 2017.

[4] Simmet A, Depa J, Tinnemann P, Stroebele-Benschop N (2017). The nutritional quality of food provided from food pantries: a systematic review of existing literature. J Acad Nutr Diet.

[5] Caspi CE, Davey C, Barsness CB, Gordon N, Bohen L, Canterbury M, Peterson H, Pratt R (2021). Needs and preferences among food pantry clients. Prev Chronic Dis.

[6] Cooksey-Stowers K, Martin KS, Schwartz M (2019). Client preferences for nutrition interventions in food pantries. J Hunger Environ Nutr.

[7] Thaler RH (2009). Sunstein CR: nudge: improving decisions about health, wealth, and happiness: Penguin.

[8] Thorndike AN, Riis J, Sonnenberg LM, Levy DE (2014). Traffic-light labels and choice architecture: promoting healthy food choices. Am J Prev Med.

[9] Levy DE, Riis J, Sonnenberg LM, Barraclough SJ, Thorndike AN (2012). Food choices of minority and low-income employees: a cafeteria intervention. Am J Prev Med.

[10] Olstad DL, Vermeer J, McCargar LJ, Prowse RJL, Raine KD (2015). Using traffic light labels to improve food selection in recreation and sport facility eating environments. Appetite.

[11] VanEpps EM, Downs JS, Loewenstein G (2016). Calorie label formats: using numeric and traffic light calorie labels to reduce lunch calories. J Public Policy Mark.

[12] Anderson E, Wei R, Liu B, Plummer R, Kelahan H, Tamez M, Marrero A, Bhupathiraju S, Mattei J. Improving healthy food choices in low-income settings in the United States using behavioral economic-based adaptations to choice architecture. Front Nutr. 2021;8:734991. 10.3389/fnut.2021.734991 PMC8526839 34692747

[13] Nudges [https://hungerandhealth.feedingamerica.org/explore-our-work/nutrition-education-initiatives/strategies/nudges/].

[14] Kelly M, Lee S-Y (2019). Diabetes and health friendly food pantry shelf design and implementation (P04-056-19). Curr Dev Nutr.

[15] Caspi CE, Canterbury M, Carlson S, Bain J, Bohen L, Grannon K, Peterson H, Kottke T (2019). A behavioural economics approach to improving healthy food selection among food pantry clients. Public Health Nutr..

[16] Wilson NLW, Just DR, Swigert J, Wansink B (2016). Food pantry selection solutions: a randomized controlled trial in client-choice food pantries to nudge clients to targeted foods. J Public Health (Oxf)..

[17] Brown L, Townsend A, Bates S. Worthington A: Using nudges to promote healthy food choices at Greater Boston Food Bank. In: *13th European Nutrition Conference,* FENS 2019, 15–18 October 2019, Malnutrition in an obese world: European perspectives. Dublin: Cambridge Core; 2020. p. 2020.

[18] Stein EC, Stowers KC, McCabe ML, White MA, Schwartz MB (2019). Ingredient bundles and recipe tastings in food pantries: a pilot study to increase the selection of healthy foods. Public Health Nutr.

[19] McKee SL, Gurganus EA, Atoloye AT, Xu R, Martin K, Schwartz MB. Pilot testing an intervention to educate and promote nutritious choices at food pantries. J Public Health (Berl). 2021.

[20] Atoloye AT, McKee SL, Gurganus EA, Xu R, Martin K, Schwartz MB (2020). P91 pilot test of supporting wellness at pantries (SWAP): clients chose healthier foods after implementation. J Nutr Educ and Behav.

[21] Cooksey-Stowers K, Martin KS, Read M, McCabe M, Cornelius T, Wolff M, Xu R, Schwartz MB (2020). Supporting Wellness at Pantries (SWAP): changes to inventory in six food pantries over one year. J Public Health (Berl)..

[22] Martin K, Xu R, Schwartz MB (2020). Food pantries select healthier foods after nutrition information is available on their food bank’s ordering platform. Public Health Nutr..

[23] Zack RM, Weil R, Babbin M, Lynn CD, Velez DS, Travis L, Taitelbaum DJ, Fiechtner L (2021). An overburdened charitable food system: making the case for increased government support during the COVID-19 crisis. Am J Public Health.

[24] Martin KS, Wolff M, Callahan K, Schwartz MB (2019). Supporting wellness at pantries: development of a nutrition stoplight system for food banks and food pantries. J Acad Nutr Diet.

[25] Healthy Eating Research Nutrition Guidelines for the Charitable Food System [https://healthyeatingresearch.org/wp-content/uploads/2020/02/her-food-bank_FINAL.pdf].

[26] Damschroder LJ, Aron DC, Keith RE, Kirsh SR, Alexander JA, Lowery JC (2009). Fostering implementation of health services research findings into practice: a consolidated framework for advancing implementation science. Implement Sci.

[27] Dedoose [Web application]. Version 9.0.17. Los Angeles: SocioCultural Research Consultants, LLC; 2021.

[28] Cooksey-Stowers K, Read M, Wolff M, Martin KS, McCabe M, Schwartz M (2019). Food pantry staff attitudes about using a nutrition rating system to guide client choice. J Hunger Environ Nutr.

[29] Thorndike AN, Gelsomin ED, McCurley JL, Levy DE (2019). Calories purchased by hospital employees after implementation of a cafeteria traffic light–labeling and choice architecture program. JAMA Netw Open.

[30] Chen HJ, Weng SH, Cheng YY, Lord AYZ, Lin HH, Pan WH (2017). The application of traffic-light food labelling in a worksite canteen intervention in Taiwan. Public Health.

[31] Scarborough P, Matthews A, Eyles H, Kaur A, Hodgkins C, Raats MM, Rayner M (2015). Reds are more important than greens: how UK supermarket shoppers use the different information on a traffic light nutrition label in a choice experiment. Int J Behav Nutr Phys Act.

[32] Hampson J, MacNell L (2022). Supporting Wellness at Pantries (SWAP) nutrition stoplight system aids rural food pantry clients living with chronic disease in selecting nutritious options. Chronic Illness..

[33] Hailemariam M, Bustos T, Montgomery B, Barajas R, Evans LB, Drahota A (2019). Evidence-based intervention sustainability strategies: a systematic review. Implement Sci.

