# Supplementary material for: A food bank program to help food pantries improve healthy food choices: mixed methods evaluation of The Greater Boston Food Bank’s Healthy Pantry Program
Source: BMC Public Health. 2023 Feb 17;23:355. doi: 10.1186/s12889-023-15243-4 (PMC9936683; doi:10.1186/s12889-023-15243-4)
Supplement: Supplementary file 1 — Additional file 1. Nutrition thresholds of green, yellow, red categories for various food groups in the 2019 Supporting Wellness at Pantries (SWAP) traffic-light nutrition ranking system. A graphic containing thresholds of saturated fat, sodium, and sugar content by food group to categorize foods into green (choose often), yellow (choose sometimes), or red (choose rarely) in the Supporting Wellness at Pantries system. [file 12889_2023_15243_MOESM1_ESM.docx]

**Additional File 1.** Nutrition thresholds of green, yellow, red categories for various food groups in the 2019 Supporting Wellness at Pantries (SWAP) traffic-light nutrition ranking system^1^


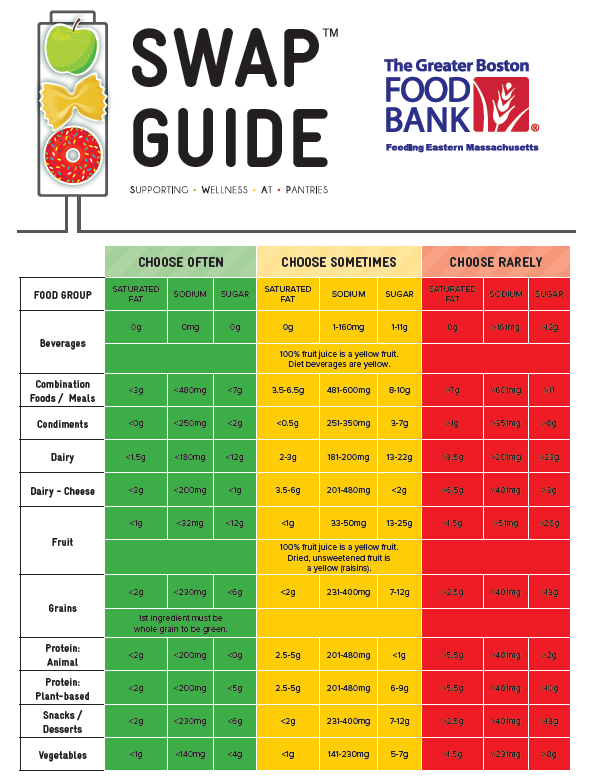


©2021 Connecticut Foodshare, Inc. All rights reserved. SWAP is a trademark of Connecticut Foodshare.

^1^SWAP was revised in 2020 with updated saturated fat, added sugar, and sodium thresholds (see *Healthy Eating Research Nutrition Guidelines for the Charitable Food System*).
