# Supplementary material for: A food bank program to help food pantries improve healthy food choices: mixed methods evaluation of The Greater Boston Food Bank’s Healthy Pantry Program
Source: BMC Public Health. 2023 Feb 17;23:355. doi: 10.1186/s12889-023-15243-4 (PMC9936683; doi:10.1186/s12889-023-15243-4)
Supplement: Supplementary file 2 — Additional file 2. Interview questionnaire administered to food pantry staff who participated in Healthy Pantry Program training. Document of interview questionnaire used for pantry staff interviews with CFIR domains. [file 12889_2023_15243_MOESM2_ESM.docx]

**Additional File 2**. Interview questionnaire administered to food pantry staff who participated in Healthy Pantry Program training

1. Before the COVID-19 pandemic, back in January or February, tell me about your agency’s involvement in Healthy Pantry Program.
2. Labeling foods in the pantry using SWAP?
3. [If Yes] Were you labeling less than half, about one half, more than a half, or all foods using SWAP?
4. Using nudges in the pantry?
5. Using Click ‘N Cook in the pantry?

**Implementation Climate**

1. (III. D. 2) Can you tell me about the process of implementing Healthy Pantry Program in your agency?

- How did it interact or conflict with other programs or processes?
- (III. D. 3) How did the priority of implementing Healthy Pantry Program compare to other priorities in your agency?

3. Initially, how long did it take for you to implement Healthy Pantry Program?

4. How much of the work involved in implementing Healthy Pantry Program was done by paid staff compared to volunteers?

5. How much of the work involved in keeping up Healthy Pantry Program after implementation was done by paid staff compared to volunteers?

6. How much staff turnover was there at your agency during the implementation period of the Healthy Pantry Program? Did it affect the implementation?

**Culture**

- 7. (III. C) How do you think your agency's characteristics (general beliefs, values, assumptions that people embrace) affected the implementation of Healthy Pantry Program?

**Evidence Strength & Quality**

- 8. (II. C) Were you aware of other agencies that had implemented Healthy Pantry Program or other similar programs?

**Access to Knowledge & Information**

9. (III. E. 3) Tell me about the guidance and education you received from the food bank.

- Did you feel the training prepared you to carry out the Healthy Pantry Program? Can you explain?
- What were positive aspects of planned training?
- What was missing from the modules that you would want?
- Additional modules? Can you suggest topics?

10. (III. E. 3) What kinds of other information and materials for Healthy Pantry Program were made available to you?

- Was it timely? Relevant? Sufficient?
- Was there any other information or materials you felt like you needed?

11**.** (III. E. 3) if you had questions about Healthy Pantry Program or its implementation, who did you ask?

- How available were these individuals?

12. (I. G) What other supports or improvements could you suggest for the delivery of Healthy Pantry Program?

- Support or working groups to share best practices and lessons learned across agencies?
- More technical assistance from GBFB?

**Cost**

13. (I. H) What costs were considered and what costs were spent when implementing Healthy Pantry Program in your agency?

**Available Resources**

14. (III. E. 2) Did you have enough resources (funding, personnel time, space, equipment, information), to run Healthy Pantry Program in the way that you had hoped?

- [If Yes] What resources did you have? Are there any other resources that you would have liked to receive?
- What resources were easy to procure?
- [If no] What resources were not available?

15. (III. E. 2) How did you procure necessary resources?

- Who was involved in helping you get what is needed?
- What challenges did you encounter?

**Sources of food**

16. How much of your pantry’s food do you usually buy from GBFB?

17. Did the amount that you order from GBFB change when you started Healthy Pantry Program? By how much?

18. Can you tell me more about the other sources of your food?

- How much say do you have in what foods you receive from these sources?
- Do you obtain food from all these sources year-round or are some sources seasonal?

19. How much does seasonality affect your agency’s ability to stock healthy foods?

20. If you ever ran out of a green food, did you ever substitute it for yellow foods when reordering/restocking? What factored into these decisions?

**Structural Characteristics**

21. (III. A) How did the infrastructure facilitate or hinder implementation of Healthy Pantry Program?

- How did you work around structural or physical challenges?

22. (III. A) What kinds of changes at your pantry were needed to accommodate Healthy Pantry Program?

- Changes in formal policies? Changes in how food is distributed? Other?

**Relative Priority**

23. (III. D. 3) How do you juggle competing priorities in your agency’s work?

- What are the other priorities?

**Learning Climate**

24. (III. D. 6) To what extent did you feel like you could try new things to improve Healthy Pantry Program in your pantry?

- 1. What were those improvements (adaptability)?
- Do you feel like you have the time and energy to think about ways to improve things?

**Champions**

25. (V. B. 3) Are there people in your agency who championed (go above and beyond what might be expected) Healthy Pantry Program?

26. (V. B. 3) What kinds of behaviors or actions did you think this individual/champion took on to help implement Healthy Pantry Program?

- For example, helping get senior leaders on board, helping solve problems? Or a small role?

**Goals & Feedback**

27. (III. D. 5) Did you/your unit/your agency set goals related to the Healthy Pantry Program?

- [If yes] What were the goals?
- How were the goals communicated in the agency? Who was informed about them?

28. (III. D. 5) To what extent were Healthy Pantry Program goals monitored for progress?

- Were changes made based on how things are going? Can you give an example?

29. (III. D. 5) Do you get any feedback about your work on Healthy Pantry Program?

**Policies & Incentives**

30. (II. D) What kind of financial or other incentives influenced the decision to implement Healthy Pantry Program?

- How did Healthy Pantry Program affect your agency's ability to receive these incentives?
- How did the Healthy Pantry Program affect payment or revenue (e.g. donations, grant funding, publicity) for your agency?
  - (III. D. 4) Were there any special recognitions or rewards or other incentives in your agency that were related to implementing Healthy Pantry Program? Can you describe them?

**Patient Needs & Resources**

31. (II. A) To what extent is staff aware of the needs and preferences of the clients being served by your agency?

32. (II. A) To what extent were the needs and preferences of the clients considered when implementing Healthy Pantry Program?

- Can you describe specific examples?
- How well was Healthy Pantry Program altered to meet their needs and preferences (E.g. use of culturally appropriate recipes, ensuring supply of culturally appropriate foods, multilingual services)?

33. (II. A) How do you think the clients responded to Healthy Pantry Program?

- Can you describe a specific story?
- Did clients mention specific barriers to using the information provided by Healthy Pantry Program?

**Adaptability for COVID**

34. During the COVID-19 pandemic, has your agency been able to continue to run the Healthy Pantry Program?

35. (I. D) What changes have you made in your pantry to adapt to the COVID-19 pandemic? (*getting food to people, changes in food supply, changes in hours or staffing*)

36. (I. D) Have you been able to continue with components of Healthy Pantry Program during the COVID-19 pandemic?

- Which components?
- What changes have you made to Healthy Pantry Program?

37. (I. D) What kind of changes or alterations could be made to Healthy Pantry Program so it will work effectively during the COVID-19 pandemic?

**Self-efficacy**

38. (IV. B) (If still using HPP) How confident are you that you will be able to continue Healthy Pantry Program at your pantry? Why?

39. (IV. B) (If stopped HPP) How confident are you that you will be able to resume Healthy Pantry Program in the future? Why?

Ask if time-permitting:

**Leadership Engagement**

1. (III. E. 1) What level of endorsement or support have you seen or heard from leaders of your pantry?
2. (III. E. 1) What level of involvement has leadership at your agency had so far with Healthy Pantry Program?

- Did they know about the intention to implement Healthy Pantry Program?
  - What kind of support do you expect going forward? Can you provide specific examples?
  - What types of barriers might they create?

**Complexity**

1. (I. F) How complex is Healthy Pantry Program?

- Please consider the following aspects of Healthy Pantry Program: *duration, scope, intricacy and number of steps involved and whether Healthy Pantry Program reflects a clear departure from previous practices*.

2. What characteristics of Healthy Pantry Program make it difficult to implement in your agency?

3. How do you think Healthy Pantry Program can be better tailored to your agency?

4. What changes to Healthy Pantry Program would make it easier to implement in your agency?

5. What changes to Healthy Pantry Program would make it easier to continue running in your agency?

**Networks & Communications**

1. (III. B) How do you typically find out about new information, such as new initiatives, accomplishments, issues, new staff, staff departures in your agency?

- What is the process of onboarding new staff?

1. (III. B) When you need to get something done or to solve a problem in your agency, who are your "go-to" people?
