# Supplementary material for: A food bank program to help food pantries improve healthy food choices: mixed methods evaluation of The Greater Boston Food Bank’s Healthy Pantry Program
Source: BMC Public Health. 2023 Feb 17;23:355. doi: 10.1186/s12889-023-15243-4 (PMC9936683; doi:10.1186/s12889-023-15243-4)
Supplement: Supplementary file 3 — Additional file 3. Demographic and work characteristics of pantry staff interviewees who had participated in Healthy Pantry Program training. A table of descriptive data on the pantry staff who were interviewed for this study. [file 12889_2023_15243_MOESM3_ESM.docx]

**Additional File 3**. Demographic and work characteristics of pantry staff interviewees who had participated in Healthy Pantry Program training

|  | **Pantry staff interviewees**  N=8  *n* (%) |
| --- | --- |
| Age in years |  |
| 25 to 44 | 3 (37.5) |
| ≥45 | 5 (62.5) |
| Gender |  |
| Female | 6 (75.0) |
| Male | 2 (25.0) |
| Race |  |
| White | 6 (75.0) |
| Prefer not to answer | 2 (25.0) |
| Hispanic | 0 (0.0) |
| Years with food pantry |  |
| ≤2 | 2 (25.0) |
| 3 to 10 | 3 (37.5) |
| ≥11 | 1 (12.5) |
| Prefer not to answer | 2 (25.0) |
| Paid staff | 3 (37.5) |
